# High-throughput single-cell analysis for the proteomic dynamics study of the yeast osmotic stress response

**DOI:** 10.1038/srep42200

**Published:** 2017-02-09

**Authors:** Rongfei Zhang, Haiyu Yuan, Shujing Wang, Qi Ouyang, Yong Chen, Nan Hao, Chunxiong Luo

**Affiliations:** 1 Center for Quantitative Biology, Academy for Advanced Interdisciplinary Studies, Peking University, Beijing, China; 2 The State Key Laboratory for Artificial Microstructures and Mesoscopic Physics, School of Physics, Peking University, Beijing, China; 3 Peking-Tsinghua Center for Life Sciences, Peking University, Beijing, China; 4 Ecole Normale Superieure, 24 rue Lhomond, 75231 Paris, France; 5 Section of Molecular Biology, Division of Biological Sciences, University of California San Diego, La Jolla, California, USA

## Abstract

Motorized fluorescence microscopy combined with high-throughput microfluidic chips is a powerful method to obtain information about different biological processes in cell biology studies. Generally, to observe different strains under different environments, high-throughput microfluidic chips require complex preparatory work. In this study, we designed a novel and easily operated high-throughput microfluidic system to observe 96 different GFP-tagged yeast strains in one switchable culture condition or 24 different GFP-tagged yeast strains in four parallel switchable culture conditions. A multi-pipette is the only additional equipment required for high-throughput patterning of cells in the chip. Only eight connections are needed to control 96 conditions. Using these devices, the proteomic dynamics of the yeast stress response pathway were carefully studied based on single-cell data. A new method to characterize the proteomic dynamics using a single cell’s data is proposed and compared to previous methods, and the new technique should be useful for studying underlying control networks. Our method provides an easy and systematic way to study signaling pathways at the single-cell level.

Cells respond to extracellular cues and adapt to new environments using intercellular signaling pathways. When cells are exposed to osmotic, oxidative and heat stresses, hundreds of proteins are involved in the response, many using their dynamic expression levels to help the cells adapt to environment. Several key points need to be addressed to study and reveal the underlying mechanisms of the signal pathway: 1. The dynamics of the signal pathway^1^,^2^; 2. Systematically analyzing the proteins involved in the control pathway^3^,^4^,^5^; and 3. Experimental and theoretical research from both the population and single-cell perspective^6^,^7^. Increasing numbers of scientists have realized that high-throughput studies of proteomic dynamics of single cells rather than steady behavior experiments are needed to determine the functions of biological networks^1^,^2^.

Several technologies have been used to study signal pathways based on the cell mRNA level, protein expression level or gene interactions, such as DNA microarrays^8^,^9^, western blot^10^ and epistatic miniarray profile (E-MAP) analysis^11^. However, these methods are limited by not using living cells, measuring the average of the cell population or having low temporal resolution. In the past decade, motorized fluorescence microscopy has become a powerful tool for chemical analysis and quantitative biological measurements because it can obtain accurate real-time images of cell morphology^12^, such as the expression level and location of proteins, cell shape, and other dynamic changes.

In these studies, microfluidic devices are often used to control the cell location, cell growth direction and micro environment to obtain high-quality cell images^13^,^14^,^15^,^16^,^17^. However, high-throughput microfluidic devices have complex construction and require complicated pipeline connections or other large, expensive machines to perform their functions^18^,^19^,^20^. Yeast cells are the model system for signaling pathway studies because of their easy genetic manipulation. For yeast mating pathway studies, Taylor *et al*. used a three polydimethylsiloxane (PDMS) layer microfluidic device that contains tens of pipeline connections to study the behavior of eight yeast strains. If Taylor’s device was modified to study the dynamics of proteins in one signaling pathway (usually one signaling pathway contains tens to hundreds of proteins), it would be too complicated because it would require hundreds of pipelines in one chip.

The *Saccharomyces cerevisiae* (budding yeast) green fluorescent protein (GFP) fusion library covering 4,159 proteins was recently used to study the DNA replication stress induced by methyl methanesulfonate (MMS)^19^. Denervaud *et al*., combined a DNA array spotter with the yeast GFP fusion library; 1,152 yeast strains were simultaneously arrayed on one coverslip. After carefully aligning the yeast strain matrix using a high-throughput microfluidic device, the yeast strains were cultured in massively parallel microchemostats. The obvious advantages, such as high-throughput, single-cell observation, and relatively high temporal resolution (20 min) provide a novel method to study the dynamic behavior of the signaling pathway. However, this method requires a complicated setup, including a DNA array spotter and aligner, and professional operation to align and bond the microfluidic device to the patterned yeast strain glass coverslip (it is very difficult to prevent the patterned dots of yeast strains from drying). However, only tens to hundreds of proteins need to be studied when we consider a specific pathway, such as the mating pathway or osmotic response pathway. For some stress responses, such as the osmotic response and oxidative response, the yeast cell responds within tens of minutes, but the time scales of most proteins in the pathways are minutes. To study these behaviors, higher temporal resolution is needed.

We developed a novel and easily operated high-throughput microfluidic system for the observation of 96 different GFP-tagged yeast strains in one switchable culture condition or 24 different GFP-tagged yeast strains in four parallel switchable culture conditions. A multi-pipette is the only additional equipment required for high-throughput patterning of cells into the chip, which makes the method easy to use in different laboratories. In this system, only eight connections are needed for 96 conditions in contrast to the hundreds of connections required for other methods. Using this system, we obtained images of 96 different conditions at 5 minute intervals in a single experiment. The high-quality images provide abundant information about hundreds of proteins in a specific pathway after treating the cells with different stimuli. The fast osmotic response behavior of budding yeast cells was carefully studied. Differences between the single-cell level and population level are discussed, and we derive more precise proteomic dynamic behavior from the single-cell data. The proteomic dynamics pattern and dynamics-based protein cluster analysis^21^ may help us to understand the underlying control mechanisms of yeast osmotic adaptation behavior.

## Results

### Device design and operation

**Figure 1:**
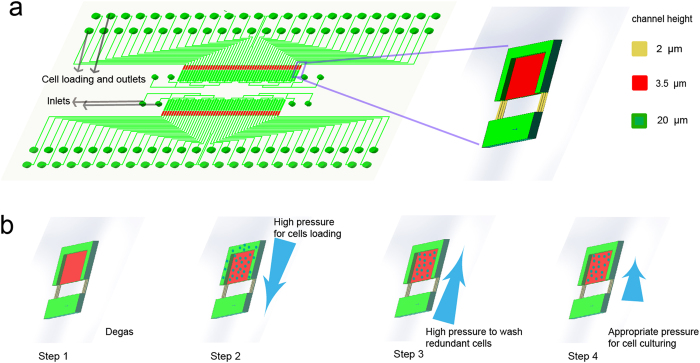
Microfluidic chip for high-throughput single-cell observation. (**a**) Chip design. (**b**) Chip application method.

We develop a conventional method to study yeast stress response pathways using a new high-throughput single-cell observation system. As Fig. 1a shows, the microfluidic device contains 4 independent cells; each cell has 24 experimental channels for different cell strains, which share two inlets for switching media. The 96 injection wells of different cell strains are arrayed as four lines on two sides of the chip separated by 3 mm. The observation areas (red parts) of different channels are arranged together in the middle of the chip. The whole chip is approximately 8 cm * 4 cm.

The right side of Fig. 1a shows an enlarged schematic image of the center area of a single channel. The green parts are used for cell loading or medium exchange. The width of the green channels is approximately 100 to 300 μm, and their height is approximately 20 μm, which is much larger than the size of a cell. The red part is the observation chamber, with a width of 200 μm and length of 300 μm. The height of the observation chambers is approximately 3.5 μm, and the yeast can be held stably for the time series images. There are multiple fences (yellow parts, 8 μm in width and 100 μm in length) between the upper and lower green channels, connecting the cell-loading/outlet channels with the medium inlet channels, whose height is 2 μm to avoid cross-contamination of yeast strains. As shown in Fig. 1b, when injecting the cell suspension from the cell-loading wells using multi-pipettes, cells can be blocked at the fences, and the yeast cells can be inserted into the observation chambers. By pumping the medium into the channels from the inlets, the cells in the green part can be removed, and the cells in the observation chamber can be retained in the devices.

**Figure 2:**
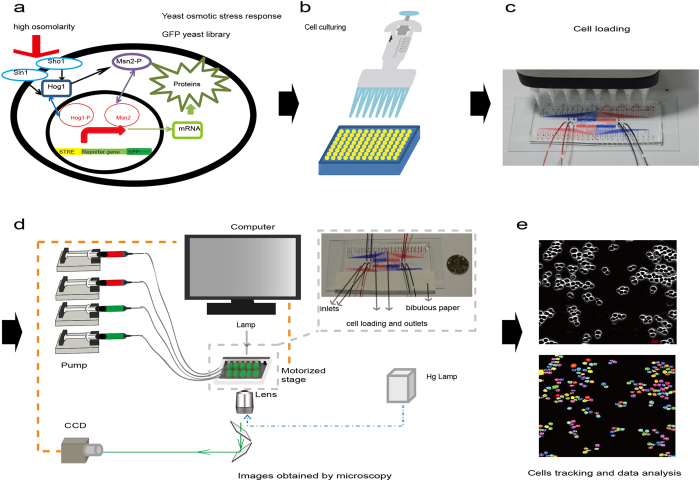
Data collection and image analysis platform. (**a**) The signal pathway for yeast osmotic stress response. (**b**) Yeast strains were cultured in 96-well plates. (**c**) Cells were loaded into the chip using an 8-channel pipette. (**d**) Images were obtained by microscope. (**e**) Data statistical analysis.

Before loading the cells, the chip is degassed in a vacuum for 20 min. The degas protocol prevents air bubbles from blocking the channels and allows the liquid to flow through the chip more easily. We then inject cells into the chip through the cell-loading wells using an 8-channel pipette, as shown in Fig. 2b and c. The tips of the 8-channel pipette are separated by 9 mm, which is three times the spacing of the cell-loading well, so we can push the strains into the chip 12 times using a preset order. After loading 96 different yeast strains into the chip within 20 min, the instantaneous flow with high pressure from the inlets washes redundant cells into the green parts, which are not located in the observation areas (red parts). Finally, supplemented medium is introduced (200 μl/h) into the chip for cell culturing (Fig. 1b). The waste liquid flows out from the cell-loading holes and evaporates from the filter paper that covers the outlets. In a relatively short time (less than 12 hours), the waste liquid is completely absorbed by the filter paper, so we can avoid connecting 96 outlet tubes.

### Protein selection from the osmotic response pathway

Yeast cells respond to osmotic stimuli through the membrane receptors Sln1 and Sho1. The transcription factors Hog1and Msn2/4 are then activated and transported into the cell nucleus^22^. More than 100 proteins downstream from the osmotic pathway are modulated by the activated Hog1 and Msn2/4 to adapt to the high osmotic environment^22^,^23^ (Fig. 2a).

Here, we select 96 important proteins, which are related to the osmotic pathway, downstream of Hog1 and Msn2/4 or related to glycerol synthesis^24^ (as shown in Supplementary Table S1), for the osmotic stimulus experiment based on a reference database and articles and test their response to 0.4 M KCl stimulus in one chip. Most of the proteins respond to osmotic stimulus on a time scale of several to tens of minutes. However, some proteins, such as transcription factors Msn2/4 and Hog1, change their location on a time scale of tens of seconds^25^. In this study, we analyze the proteomic dynamic behavior with a temporal resolution of 5 min, so we delete some rapidly changing proteins from our primary selections. To study both the enhanced and decreased behavior of the protein expression level at different osmotic stimuli levels (0 M, 0.2 M, 0.4 M, 0.8 M KCl), we select 40 proteins from the initial proteins with high SNR (signal to noise ratio >7) and large fold change (fold change greater than 1.2) for the continuous experiments and data analysis. Their functions are described in Supplementary Table S2^26^,^27^,^28^,^29^,^30^,^31^,^32^,^33^,^34^.

The selected proteins include some well-known proteins: GPH1, a glycogen phosphorylase, is required for the mobilization of glycogen, and its expression is regulated by stress-response elements and by the HOG1 mitogen-activated protein kinase (MAPK) pathway; HSP12, a plasma membrane protein, is involved in maintaining membrane organization and maintaining organization during stress conditions; GRE2, is stress induced (osmotic, ionic, oxidative, heat shock and heavy metals) and is regulated by the HOG pathway; and TDH1 is involved in glycolysis and gluconeogenesis.

### Quantifying the proteomic dynamic behavior under different KCl stimulus concentrations

We can obtain images from 96 points and two channels (phase contrast channel and fluorescence channel) using the microscope system (Fig. 2d). First, we use ImageJ to transform the phase contrast images to mask pictures. Then, we use the cell tracker software provided by Nan Hao’s group to track single cells^35^. Using the traced mask figures, we can read out the GFP fused protein expression and location information at the single-cell level (Fig. 2e). For proteins that are uniformly distributed in the cell or located at the membrane or mitochondria, we use the mean intensity of the fluorescence signals of the whole cell to represent the protein concentration. For proteins located in the nucleus, we use the mean of the top 10% fluorescence signals to represent the protein concentration.

**Figure 3:**
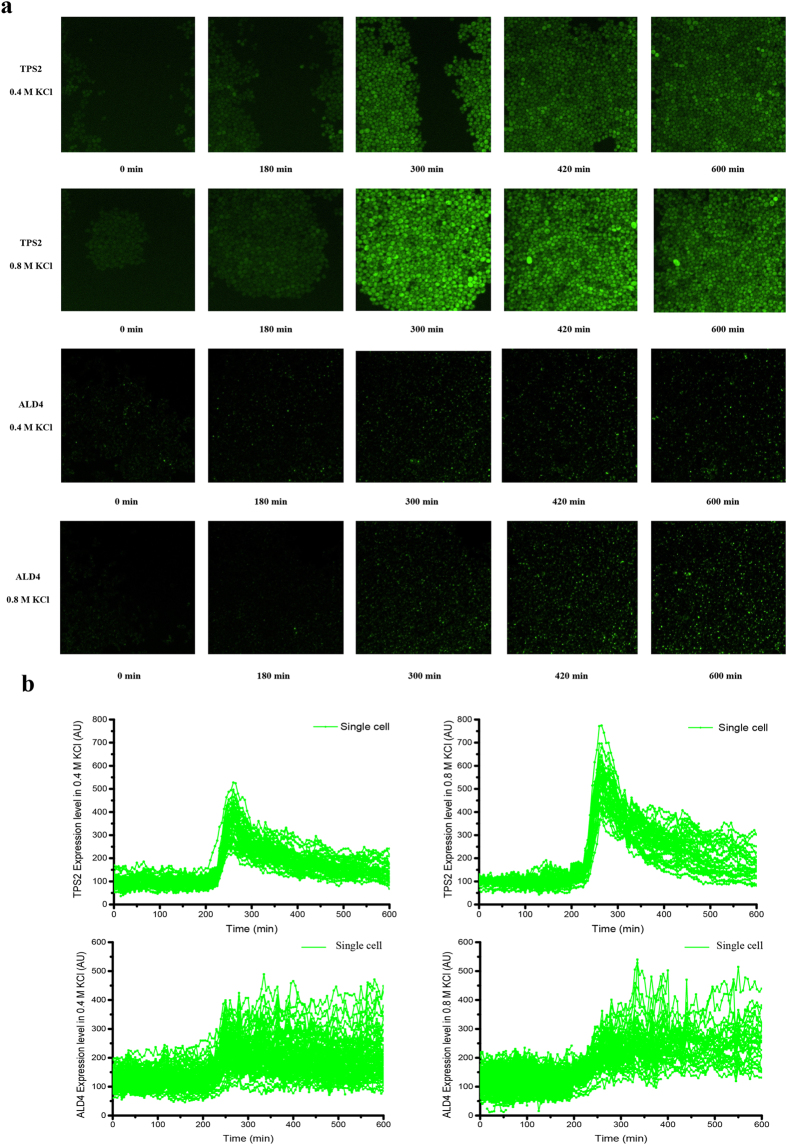
Original fluorescence images at the single-cell level. (**a**) The original fluorescence images of TPS2 and ALD4 (0.4 M KCl or 0.8 M KCl was added at 180 min). (**b**) TPS2 and ALD4 expression in osmotic stress response at the single-cell level.

Figure 3a shows time lapse images of two strains: the fluorescence signal of TPS2 is almost uniformly distributed in the cell and the fluorescence signal of ALD4 is located in the mitochondria. After carefully removing the background signal and correcting the photobleaching effect, the fluorescence signals of single cells can be plotted as a function of time, as shown in Fig. 3b.

**Figure 4:**
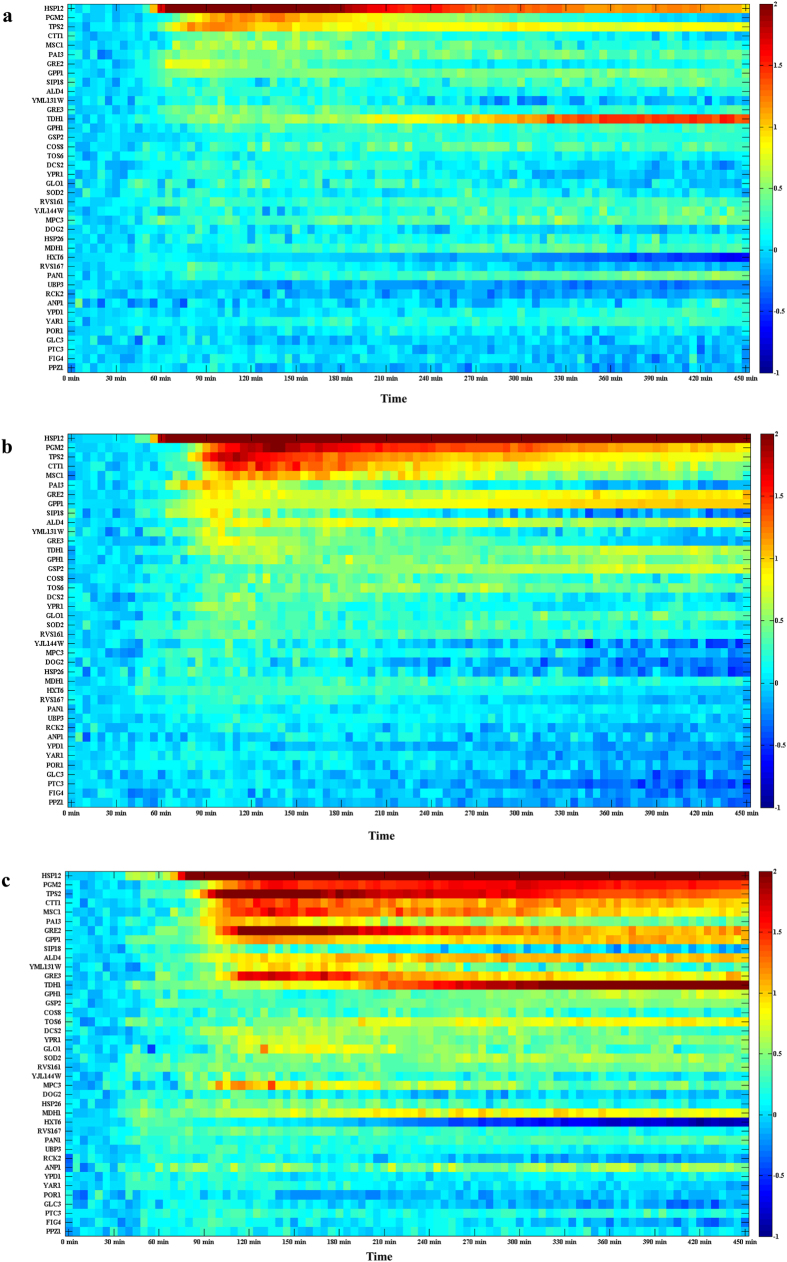
The protein expression dynamics under osmotic stress (log base 2 of the normalized expression level). (**a**) 0.2 M KCl, (**b**) 0.4 M KCl, (**c**) 0.8 M KCl. The stimuli were added at 30 min.

We explore the behavior of 24 proteins stimulated with 0, 0.2, 0.4, or 0.8 M KCl using a single chip, analyzing 40 proteins in two separate experiments. Eight common proteins are studied in the two experiments to confirm that the results are comparable. We repeat the experiment at least twice and obtain similar results (Supplementary Fig. S1). Although we can simultaneously obtain tens to hundreds of single-cell data from one chamber, the number of single-cell trajectories from beginning to end is approximately tens because there are only 20–50 cells initially and some of the cells are extruded from the observation chamber and washed to the outlet during cell growth. Therefore, we use the average fluorescence signal of all cells in one chamber as a function of time to characterize the dynamic response to osmotic stimulus at the population level with temporal resolution of 5 min. Each time point has at least tens of cells to be averaged. We plot the heat map of the protein responses (log base 2 of the normalized expression level) as a function of time for cells treated with different concentrations of KCl, as shown in Fig. 4. The data are normalized with the initial 0.5 hour when the cells are cultured in synthetic complete (SC) medium without histidine (His^-^). Therefore, we can easily determine the proteomic dynamic changes after treatment with KCl. Most of the proteins in our research are upregulated, but some are downregulated.

Some proteins, such as TPS2, MSC1, ALD4, GRE2, GRE3, MDH1, GPP1 and YML131W, significantly adjust their expression level at different stimuli levels. GRE2, GRE3, MDH1, and GPP1 also adjust their response time scale at different stimuli levels (Supplementary Fig. S2). The heat map and comparison of the proteomic dynamic behavior under different stimuli conditions provide information about the protein network. Therefore, using the simple method, we determine the proteomic dynamic pattern of the osmotic signaling pathway at different stimuli levels. The dynamic curves of the well-known proteins from our method are consistent with previous results from other groups^36^,^37^,^38^. These relevant studies were mostly conducted using biochemistry methods, such as western blot, luciferase assays, immunofluorescence and RNA-seq, so their results did not have much temporal information and only evaluated the average of the population. However, we carefully compared their results with our data. For example, GPP1, PGM2, and CTT1 increased their expression levels markedly, similarly to the transcriptional response in the previous work^39^,^40^. GRE2 reached its maximum expression level approximately 40–60 min after adding KCl and then rapidly adapted^41^. POR1 changed its expression level slightly in response to osmotic stress^42^.

Although the protein expression dynamics of the cells at the population level provide information of how the cells respond to osmotic stimulus, it is unclear whether using the average of all the cells to characterize the dynamics of proteins can reveal the real dynamic process. Previous reports and reviews show that some proteomic dynamics at the single-cell level are not consistent with the population level, especially when the protein expression level in the single cell oscillates or increases at different time points^3^.

To obtain more long trajectories of single cells and to systematically compare the differences in proteomic dynamic behavior between the single-cell and population levels, we repeat the 0.4 M KCl stimulus experiment with all 40 strains twice in one chip. Therefore, we can perform four repeated experiments with 0.4 M KCl stimulus for all 40 strains, including the previous experiments. For each strain, we obtain more than 30 long trajectories of single cells for further analysis.

### Proteomic dynamic curves at the single-cell level

**Figure 5:**
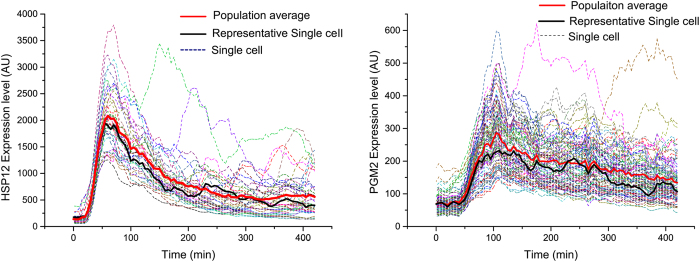
Single-cell analysis of the protein expression patterns. (**a**) Single-cell analysis of HSP1; (**b**) Single-cell analysis of PGM2. The stimuli were added at 0 min.

Although the dynamic curves of proteins show similar characteristics for the cells in the same chamber, the response time, the protein produce rates and steady state levels of some proteins vary (Fig. 5). Hsp12 belongs to the family of stress proteins, and in the previous research, bursts in Hsp12 protein levels were observed^43^. However, we do not observe bursts at the population level.

Therefore, we attempt to find the proteomic dynamics in a single cell, that is most similar to the other cells, to represent the dynamic curve of the protein. We define parameter **M1** (eq. (1)) to measure the similarity of one cell to the others and identify the single cell with the minimum **M1**.


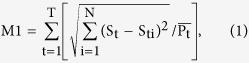


where S_t_ is the protein expression level of a single cell, and S_ti_ is the protein expression level of another cell. 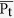 is the mean value of all cells’ protein expression levels at a specific time. N is the total cell number, and T is the total number of time points. Therefore, the proteomic dynamics of the cell with the minimum M1 represents the proteomic dynamics at the single-cell level.

Similarly, we define parameter **M2** (eq. (2)) to measure the difference between the single-cell data and the population result.


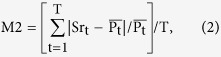


where Sr_t_ is the protein expression level of the representative single cell with the minimum M1, and 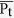 is the protein expression at the population level. We calculate M2 for all forty proteins (Supplementary Table S3) and find that HSP12 and PGM2 have higher M2 than the other proteins (Fig. 5). The dynamics of the two proteins show small bursts after the first peak response to osmotic stimulation. Bursts of Hsp12 were found in previous research^43^ but are not observed at the population level, which means the proteomic dynamics obtained by our method at the single-cell level are more accurate than the population level. Therefore, our method is useful and powerful. Additionally, we use one quantitative parameter (M2) to measure the single-cell variance, and it is important for us to accurately determine the dynamics of the proteins.

The protein expression levels of many proteins involved in stress response with relatively high M2, such as GPH1, GRE2, TDH1, and PAI3, were studied by western blot but have not been studied at the single-cell level^26^,^27^,^28^. Our method will benefit these studies.

### Cluster analysis using proteomic dynamic data at the single-cell level

**Figure 6:**
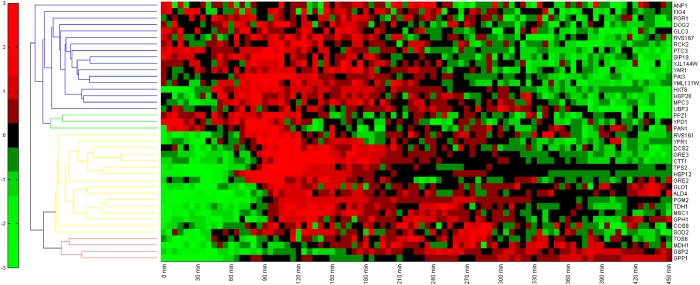
Cluster analysis using the proteomic dynamic data from a single cell (log base 2 of the normalized expression level in 0.4 M KCl; the stimuli were added at 30 min).

We obtain all the proteomic dynamic curves from single cells with 0.4 M KCl stimulus. Although there may be many mathematic methods to analyze our data, we cluster the proteins using their dynamic behavior, as shown in Fig. 6, using the Matlab clustergram function. The rows of the row-clustered data indicate that the behavior of the forty proteins is automatically clustered into four main modules.

The first module, shown in blue, is rapidly upregulated and then adapts. The second module, shown in green, is downregulated. The third module, shown in yellow, is slowly upregulated and then adapts. The fourth module, shown in red, is upregulated after a long time without adaptation. The proteomic dynamics in all modules may have bursts in their response. The behaviors in more downstream rows represent behaviors between two related proteins that are similar, which may indicate that the two proteins are close to each other or have the same function. GPH1, TDH1, GRE2 and GRE3 are glycerol metabolizing or synthesizing proteins; they are all clustered in the same module. HSP26, SIP18, RCK2 and DOG2 are stress response proteins; they are all clustered in the same module.

In the future, dealing with a larger control network and the interesting dynamic behavior of proteins, the dynamic cluster method using dynamic data from a single cell obtained using our system may simplify the control network structures and reveal the underlying mechanisms.

## Discussion and Conclusion

We study proteomic dynamic behavior under osmotic stress at the single-cell level using our system. The temporal resolution of our method for 96 conditions is as high as 5 minutes, which is not only better than the temporal resolution of traditional DNA/RNA chip technology but also better than that of high-throughput flow cytometry methods. In addition, we show that the data obtained from time lapse single-cell trajectories may provide more important information than data from the population level.

Additionally, our method is different from other high-throughput microfluidic devices based on several advantages, such as no large equipment is required for cell loading; fewer connections are needed for our high-throughput chip; in addition, the system setup is much easier and reliable. Therefore, our system could be easily extended for use by biologists and physicists.

However, there are some disadvantages tousing our method to study signaling pathways, such as our methods cannot analyze protein methylation or phosphorylation processes if the protein does not change its location or expression level when treated with stimuli; and our protocol uses the 5 min temporal interval to image the protein dynamics, which is not efficient to study rapidly changing proteins, such as some transcription factors.

Based on the advantages and disadvantages of our system, our method is not limited to study stress pathways of budding yeast but is also suitable for studying other signaling pathways in many biology laboratories. We propose a new method to use the main characteristics of single-cell dynamic behavior, which may be useful for the analysis of protein dynamic processes with large variations at the single-cell level. The data obtained from our system should be useful for future network reconstructions.

## Methods

### Chip fabrication

The chip mold was fabricated using standard three-layer lithography with Su8 photoresist (MicroChem, US). The PDMS layer was fabricated using standard soft-lithography techniques. After the solidified PDMS layer (6–8 mm thickness) was peeled from the mold, the cell-loading wells and medium inlets were punched using a sharp metal puncher with a 1 mm diameter. Then, the PDMS layer was bonded to a special-ordered coverglass (10 cm * 5 cm * 0.17 mm) using air plasma.

### Preparation of yeast cells

The *Saccharomyces cerevisiae* GFP fusion library was generated by Dr. Erin O’Shea and Dr. Jonathan Weissman at UCSF. The library covers 4,159 strains with different proteins fused with *Aequorea Victoria* GFP protein without affecting their functions. For each experiment, we replicated a different subset of yeast-GFP strains into 96-well plates (P-DW-20-CS, AXYGEN). To prevent cross-contamination, the plate was sealed with a breathable adhesive membrane (BF-400 SEALING FILM, AXYGEN). After incubation for 16–20 hours at 30 °C and 220 rpm in SC medium His^-^, the cell suspensions were diluted into another 96-well plate by 10 times and cultured for an additional 4 hours before use.

### Microfluidic device and microscope setup

After the chip was connected to the syringe pumps, the chip was placed on an automated fluorescence microscope (Nikon Ti-E). A total of 96 preset observation points were determined from three observation points at the boundaries using a coordinate calculator. Each observation point was imaged in phase contrast and fluorescence at 40*1.5X magnification with 5 minute resolution. After culturing the cells on the chip for the first three hours under SC medium His^-^, the medium was switched to SC medium His^-^ with different concentrations of KCl for an additional seven hours. In total, 10 hours of video at 96 points from two channels (phase contrast channel and fluorescence channel) were obtained, and each point contained tens of cells at the beginning and hundreds of cells at the end (almost filling the observation area). The growth rate of the yeast was nearly the same as previous studies^16^ when cultured in our device, which means the exchange of medium is fast enough for cell growth under the exponential growth state.

## Additional Information

**How to cite this article:** Zhang, R. *et al*. High-throughput single-cell analysis for the proteomic dynamics study of the yeast osmotic stress response. *Sci. Rep.* **7**, 42200; doi: 10.1038/srep42200 (2017).

**Publisher's note:** Springer Nature remains neutral with regard to jurisdictional claims in published maps and institutional affiliations.

## Supplementary Material

Supplementary Information
