# Supplementary material for: High-throughput single-cell analysis for the proteomic dynamics study of the yeast osmotic stress response
Source: Sci Rep. 2017 Feb 9;7:42200. doi: 10.1038/srep42200 (PMC5299847; doi:10.1038/srep42200)
Supplement: Supplementary Information [file srep42200-s1.pdf]

SUPPLEMENTARY INFORMATION

High-throughput single-cell analysis for the proteomic dynamics study of the yeast osmotic stress response

Rongfei Zhang[1], Haiyu Yuan[2], Shujing Wang[1], Qi Ouyang[1,2,3], Yong Chen[4], Nan Hao[5], Chunxiong Luo[1,2*]

[1]Center for Quantitative Biology, Academy for Advanced Interdisciplinary Studies, Peking University, Beijing, China

[2]The State Key Laboratory for Artificial Microstructures and Mesoscopic Physics, School of Physics, Peking University, Beijing, China

[3]Peking-Tsinghua Center for Life Sciences, Peking University, Beijing, China

[4]Ecole Normale Superieure, 24 rue Lhomond, 75231 Paris, France

[5]Section of Molecular Biology, Division of Biological Sciences, University of California San Diego, La Jolla, California, USA.

[*]To whom correspondence should be addressed: ChunxiongLuo, Email: pkuluocx@pku.edu.cn, Tel.: +86-10-62754743

Supplementary Table S1 96 important proteins for the osmotic stimuli experiment

Supplementary Table S2 The functions of 40 proteins from the primary selected proteins

Supplementary Table S3 The M2 values of the 40 proteins.

Supplementary Fig. S1 The dynamic behaviors of SIP18 and PAI3 in different experiments (Test A and Test B). The stimuli (0.4 M KCl) are added at 180 min.

Supplementary Fig. S2 The dynamic behaviors of (**a**) TPS2, **(b)** GPP1, (**c**) GRE2, and **(d)** MDH1 with different stimuli levels. The stimuli are added at 180 min.

**1**	**UBP3**	**25**	**DOG2**	49	ALD3	73	SNF1
**2**	**FIG4**	**26**	**HSP26**	50	TKL2	74	GAL11
**3**	**PPZ1**	**27**	**SOD2**	51	RTN2	75	RIM15
**4**	**GPH1**	**28**	**YAR1**	52	DDR2	76	MSN5
**5**	**GLC3**	**29**	**YPD1**	53	HXK1	77	TPK2
**6**	**TPS2**	**30**	**PAI3**	54	SKN7	78	YAK1
**7**	**RCK2**	**31**	**YJL144W**	55	GPD1	79	GCN5
**8**	**PGM2**	**32**	**SIP18**	56	FPS1	80	GIS1
**9**	**CTT1**	**33**	**MPC3**	57	AQY2	81	PSR1
**10**	**RVS167**	**34**	**TOS6**	58	GON7	82	PSR2
**11**	**PTC3**	**35**	**HSP12**	59	GRE1	83	MSN4
**12**	**MSC1**	**36**	**PAN1**	60	HLR1	84	SNF2
**13**	**ALD4**	**37**	**ANP1**	61	OSM1	85	RIM11
**14**	**DCS2**	**38**	**HXT6**	62	PBS2	86	YGK3
**15**	**COS8**	**39**	**GSP2**	63	POR2	87	SRB10
**16**	**YML131W**	**40**	**POR1**	64	PPZ2	88	CYR1
**17**	**YPR1**	41	HXT7	65	SKO1	89	TOR2
**18**	**GRE2**	42	USV1	66	SMP1	90	CDC25
**19**	**GRE3**	43	YGP1	67	SSK2	91	EAF7
**20**	**GLO1**	44	YNL300W	68	STL1	92	RAS2
**21**	**TDH1**	45	MCR1	69	RPD3	93	MSN2
**22**	**MDH1**	46	DDR48	70	MCK1	94	HOG1
**23**	**GPP1**	47	MMF1	71	HDA3	95	MSN1
**24**	**RVS161**	48	RLB5	72	WHI2	96	WHI5

Supplementary Table S1 96 important proteins for the osmotic stimuli experiment

1	UBP3	Ubiquitin-specific protease involved in transport and osmotic response; negatively regulates Ras/PKA signaling; interacts with Bre5p to coregulate anterograde, retrograde transport between ER and Golgi; involved in transcription elongation in response to osmostress through. phosphorylation at Ser695 by Hog1p; inhibitor of gene silencing; role in ribophagy; cleaves ubiquitin fusions but not polyubiquitin; protein abundance increases in response to DNA replication stress.
2	FIG4	Phosphatidylinositol 3,5-bisphosphate (PtdIns[3,5]P) phosphatase; required for efficient mating and response to osmotic shock; physically associates with and regulated by Vac14p; contains a SAC1-like domain; homologous to human FIG4, which is associated with CMT4J, a form of Charcot-Marie-Tooth disorde .
3	PPZ1	Serine/threonine protein phosphatase Z, isoform of Ppz2p; involved in regulation of potassium transport, which affects osmotic stability, cell cycle progression, and halotolerance.
4	GPH1	Glycogen phosphorylase required for the mobilization of glycogen; non-essential; regulated by cyclic AMP-mediated phosphorylation; expression is regulated by stress-response elements and by the HOG MAP kinase pathway.
5	GLC3	Glycogen branching enzyme, involved in glycogen accumulation; green fluorescent protein (GFP)-fusion protein localizes to the cytoplasm in a punctate pattern; relocalizes from nucleus to cytoplasmic foci upon DNA replication stress; glycogen accumulation defect of the null mutant is functionally complemented by human GBE1, which is associated with glycogen storage disease.
6	TPS2	Phosphatase subunit of the trehalose-6-P synthase/phosphatase complex; involved in synthesis of the storage carbohydrate trehalose; expression is induced by stress conditions and repressed by the Ras-cAMP pathway; protein abundance increases in response to DNA replication stress.
7	RCK2	Protein kinase involved in response to oxidative and osmotic stress; identified as suppressor of S. pombe cell cycle checkpoint mutations; similar to CaM (calmodulin) kinases; RCK2 has a paralog, RCK1, that arose from the whole genome duplication.

8	PGM2	Phosphoglucomutase; catalyzes the conversion from glucose-1-phosphate to glucose-6-phosphate, which is a key step in hexose metabolism; functions as the acceptor for a Glc-phosphotransferase; protein abundance increases in response to DNA replication stress; PGM2 has a paralog, PGM1, that arose from the whole genome duplication.
9	CTT1	Cytosolic catalase T; has a role in protection from oxidative damage by hydrogen peroxide 3.
10	RVS167	Actin-associated protein with roles in endocytosis and exocytosis; N-BAR domain protein that interacts with Rvs161p to regulate actin cytoskeleton, endocytosis, and viability following starvation or osmotic stress; recruited to bud tips by Gyl1p and Gyp5p during polarized growth; homolog of mammalian amphiphysin.
11	PTC3	ype 2C protein phosphatase (PP2C); dephosphorylates Hog1p (see also Ptc2p) to limit maximal kinase activity induced by osmotic stress; dephosphorylates T169 phosphorylated Cdc28p (see also Ptc2p); role in DNA damage checkpoint inactivation; PTC3 has a paralog, PTC2, that arose from the whole genome duplication.
12	MSC1	Protein of unknown function; mutant is defective in directing meiotic recombination events to homologous chromatids; the authentic, non-tagged protein is detected in highly purified mitochondria and is phosphorylated.
13	ALD4	Mitochondrial aldehyde dehydrogenase; required for growth on ethanol and conversion of acetaldehyde to acetate; phosphorylated; activity is K+ dependent; utilizes NADP+ or NAD+ equally as coenzymes; expression is glucose repressed; can substitute for cytosolic NADP-dependent aldehyde dehydrogenase when directed to the cytosol; human homolog ALDH2 can complement yeast ald4 mutant.
14	DCS2	m(7)GpppX pyrophosphatase regulator; non-essential, stress induced regulatory protein; modulates m7G-oligoribonucleotide metabolism; inhibits Dcs1p; regulated by Msn2p, Msn4p, and the Ras-cAMP-cAPK signaling pathway; mutant has increased aneuploidy tolerance; DCS2 has a paralog, DCS1, that arose from the whole genome duplication.

15	COS8	Endosomal protein involved in turnover of plasma membrane proteins; member of the DUP380 subfamily of conserved, often subtelomeric COS genes; required for the multivesicular vesicle body sorting pathway that internalizes plasma membrane proteins for degradation; Cos proteins provide ubiquitin in trans for nonubiquitinated cargo proteins.
16	YML131W	Protein of unknown function; similar to medium chain dehydrogenase/reductases; expression induced by stresses including osmotic shock, DNA damaging agents, and other chemicals; GFP-fusion protein localizes to the cytoplasm; protein abundance increases in response to DNA replication stress.
17	YPR1	NADPH-dependent aldo-keto reductase; reduces multiple substrates including 2-methylbutyraldehyde and D,L-glyceraldehyde, expression is induced by osmotic and oxidative stress; functionally redundant with other aldo-keto reductases; protein abundance increases in response to DNA replication stress; YPR1 has a paralog, GCY1, that arose from the whole genome duplication; human homolog AKR1B1 can complement yeast null mutant.
18	GRE2	3-methylbutanal reductase and NADPH-dependent methylglyoxal reductase; stress induced (osmotic, ionic, oxidative, heat shock and heavy metals); regulated by the HOG pathway; restores resistance to glycolaldehyde by coupling reduction of glycolaldehyde to ethylene glycol and oxidation of NADPH to NADP+; protein abundance increases in response to DNA replication stress; methylglyoxal reductase (NADPH-dependent) is also known as D-lactaldehyde dehydrogenase.
19	GRE3	Aldose reductase; involved in methylglyoxal, d-xylose, arabinose, and galactose metabolism; stress induced (osmotic, ionic, oxidative, heat shock, starvation and heavy metals); regulated by the HOG pathway; protein abundance increases in response to DNA replication stress.
20	GLO1	Monomeric glyoxalase I; catalyzes the detoxification of methylglyoxal (a by-product of glycolysis) via condensation with glutathione to produce S-D-lactoylglutathione; expression regulated by methylglyoxal levels and osmotic stress.

21	TDH1	Glyceraldehyde-3-phosphate dehydrogenase (GAPDH), isozyme 1; involved in glycolysis and gluconeogenesis; tetramer that catalyzes the reaction of glyceraldehyde-3-phosphate to 1,3 bis-phosphoglycerate; detected in the cytoplasm and cell wall; protein abundance increases in response to DNA replication stress; GAPDH-derived antimicrobial peptides secreted by S. cerevisiae are active against a wide variety of wine-related yeasts and bateria 1.
22	MDH1	Mitochondrial malate dehydrogenase; catalyzes interconversion of malate and oxaloacetate; involved in the tricarboxylic acid (TCA) cycle; phosphorylated.
23	GPP1	Constitutively expressed DL-glycerol-3-phosphate phosphatase; also known as glycerol-1-phosphatase; involved in glycerol biosynthesis, induced in response to both anaerobic and osmotic stress; GPP1 has a paralog, GPP2, that arose from the whole genome duplication.
24	RVS161	Amphiphysin-like lipid raft protein; N-BAR domain protein that interacts with Rvs167p and regulates polarization of the actin cytoskeleton, endocytosis, cell polarity, cell fusion and viability following starvation or osmotic stress.
25	DOG2	2-deoxyglucose-6-phosphate phosphatase; member of a family of low molecular weight phosphatases, induced by oxidative and osmotic stress, confers 2-deoxyglucose resistance when overexpressed; DOG2 has a paralog, DOG1, that arose from a single-locus duplication; the last half of DOG1 and DOG2 are subject to gene conversions among S. cerevisiae, S. paradoxus, and S. mikatae.
26	HSP26	Small heat shock protein (sHSP) with chaperone activity; forms hollow, sphere-shaped oligomers that suppress unfolded proteins aggregation; long-lived protein that is preferentially retained in mother cells and forms cytoplasmic foci; oligomer activation requires heat-induced conformational change; also has mRNA binding activity 3.
27	SOD2	Mitochondrial manganese superoxide dismutase; protects cells against oxygen toxicity.
28	YAR1	Ankyrin-repeat containing, nucleocytoplasmic shuttling chaperone; prevents aggregation of Rps3p in the cytoplasm, associates with nascent Rps3p during its

		translation in the cytoplasm and delivers it to the 90S in the nucleus; required for 40S ribosomal subunit export, biogenesis and adaptation to osmotic and oxidative stress; expression repressed by heat shock.
29	YPD1	Osmotic stress-responsive phosphorelay intermediate sensor protein; phosphorylated by the plasma membrane sensor Sln1p in response to osmotic stress and then in turn phosphorylates the response regulators Ssk1p in the cytosol and Skn7p in the nucleus.
30	PAI3	Cytoplasmic proteinase A (Pep4p) inhibitor; dependent on Pbs2p and Hog1p protein kinases for osmotic induction; intrinsically unstructured, N-terminal half becomes ordered in the active site of proteinase A upon contact.
31	YJL144W	Cytoplasmic hydrophilin essential in desiccation-rehydration process; expression induced by osmotic stress, starvation and during stationary phase; protein abundance increases in response to DNA replication stress 1.
32	SIP18	Phospholipid-binding hydrophilin; essential to overcome desiccation-rehydration process; expression is induced by osmotic stress; SIP18 has a paralog, GRE1, that arose from the whole genome duplication.
33	MPC3	Highly conserved subunit of the mitochondrial pyruvate carrier (MPC); expressed during growth on nonfermentable carbon sources, and heterodimerizes with Mpc1p to form the respiratory isoform of MPC; MPC localizes to the mitochondrial inner membrane and mediates pyruvate uptake; MPC3 paralog, MPC2, heterodimerizes with Mpc1p to form the fermentative MPC isoform; protein abundance increases in response to DNA replication stress.
34	TOS6	Glycosylphosphatidylinositol-dependent cell wall protein; expression is periodic and decreases in respone to ergosterol perturbation or upon entry into stationary phase; depletion increases resistance to lactic acid.
35	HSP12	Plasma membrane protein involved in maintaining membrane organization; involved in maintaining organization during stress conditions; induced by heat shock, oxidative stress, osmostress, stationary phase, glucose depletion, oleate and alcohol; protein abundance increased in response to DNA replication stress and

		dietary restriction; regulated by the HOG and Ras-Pka pathways; required for dietary restriction-induced lifespan extension.
36	PAN1	Part of actin cytoskeleton-regulatory complex Pan1p-Sla1p-End3p; associates with actin patches on cell cortex; promotes protein-protein interactions essential for endocytosis; binds to and activates Arp2/3 complex in vitro; phosphorylation of Thr-1225 is regulated by MAPK Hog1p in response to osmotic stress; previously thought to be a subunit of poly(A) ribonuclease.
37	ANP1	Subunit of the alpha-1,6 mannosyltransferase complex; type II membrane protein; has a role in retention of glycosyltransferases in the Golgi; involved in osmotic sensitivity and resistance to aminonitrophenyl propanediol.
38	HXT6	High-affinity glucose transporter; member of the major facilitator superfamily, nearly identical to Hxt7p, expressed at high basal levels relative to other HXTs, repression of expression by high glucose requires SNF3; HXT6 has a paralog, HXT1, that arose from the whole genome duplication.
39	GSP2	GTP binding protein (mammalian Ranp homolog); involved in the maintenance of nuclear organization, RNA processing and transport; interacts with Kap121p, Kap123p and Pdr6p (karyophilin betas); not required for viability; protein abundance increases in response to DNA replication stress; GSP2 has a paralog, GSP1, that arose from the whole genome duplication 1.
40	POR1	Mitochondrial porin (voltage-dependent anion channel); outer membrane protein required for maintenance of mitochondrial osmotic stability and mitochondrial membrane permeability; couples the glutathione pools of the intermembrane space (IMS) and the cytosol; interacts with Om45 and Om14 in the outer membrane; phosphorylated; protein abundance increases in response to DNA replication stress.

Supplementary Table S2 The functions of 40 proteins from the primary selected proteins

Gene	M2	Gene	M2
HSP12	0.15312	GLO1	0.050379
PGM2	0.124257	YPR1	0.050098
GRE2	0.123721	ANP1	0.049418
TDH1	0.121697	RVS161	0.047604
GPH1	0.092405	PPZ1	0.047256
TPS2	0.08436	UBP3	0.046457
PAI3	0.072141	GRE3	0.045428
SIP18	0.069776	DCS2	0.044157
CTT1	0.067589	YPD1	0.042697
ALD4	0.061341	MPC3	0.041139
MDH1	0.060178	FIG4	0.039532
GSP2	0.055119	SOD2	0.039479
YML131W	0.054998	YJL144W	0.039278
TOS6	0.053963	POR1	0.038654
HSP26	0.053951	PTC3	0.037191
MSC1	0.053087	YAR1	0.03599
DOG2	0.052952	HXT6	0.035307
GPP1	0.052837	COS8	0.034797
PAN1	0.050747	RVS167	0.033569
GLC3	0.05074	RCK2	0.031353

Supplementary Table S3 The M2 values of the 40 proteins.

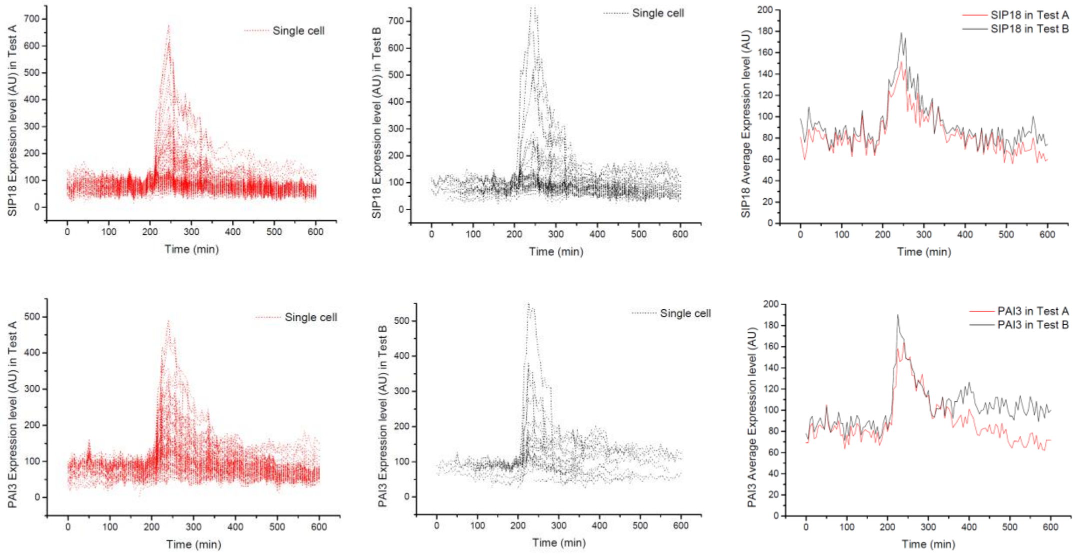

Supplementary Fig. S1 The dynamic behaviors of SIP18 and PAI3 in different experiments (Test A and Test B). The stimuli (0.4 M KCl) are added at 180 min.

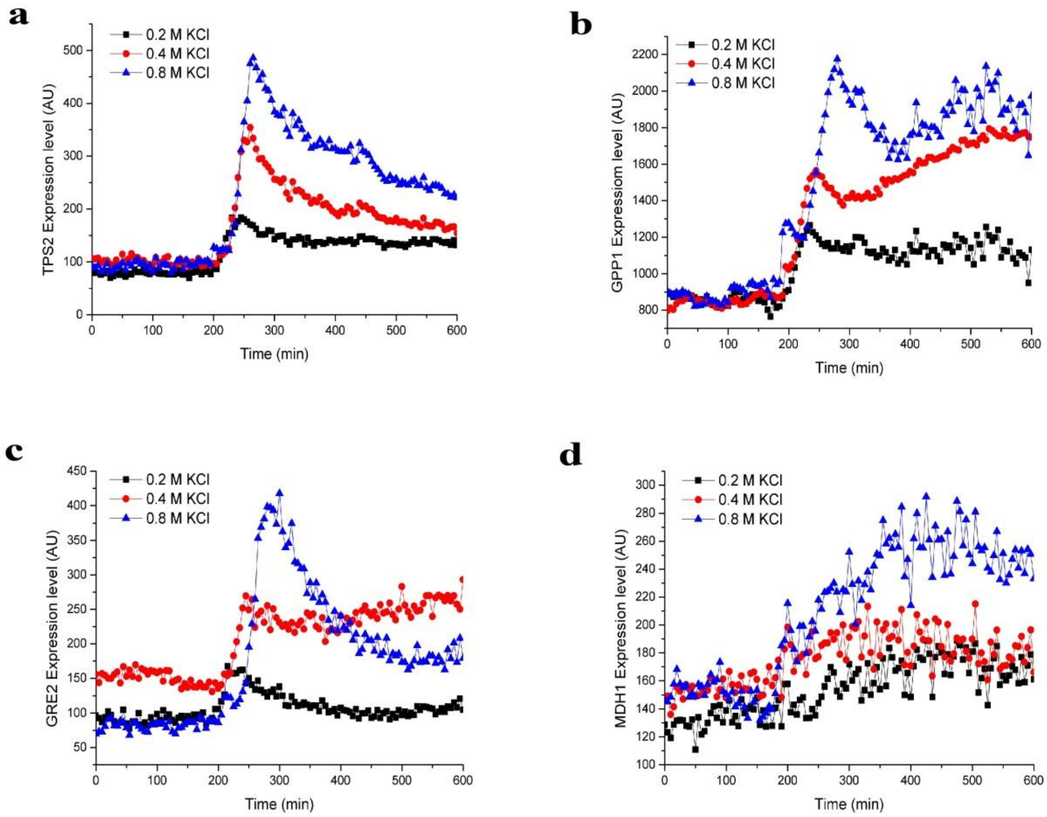

Supplementary Fig. S2 The dynamic behaviors of (**a**) TPS2, **(b)** GPP1, (**c)** GRE2, and **(d)** MDH1 with different stimuli levels. The stimuli are added at 180 min.
